# Long-term weight loss of distal gastric bypass is moderately superior compared to proximal gastric bypass in patients with a BMI of 37–44 Kg/m^2^

**DOI:** 10.1007/s00423-024-03348-2

**Published:** 2024-05-21

**Authors:** Teresa Cereser, Jan Heil, Othmar Schöb, Rolf Schlumpf, Walter A. Gantert, David Infanger, Michael Böckmann, Philippe Beissner, Birgit Bach-Kliegel, Natascha Potoczna, Marc Schiesser

**Affiliations:** 1 Chirurgisches Zentrum Zürich, Klinik Hirslanden, Zurich, Switzerland; 2 Klinik für Allgemein-, Viszeral-, Transplantation- and Thoraxchirurgie, Goethe-Universität Frankfurt, Universitätsklinikum, Frankfurt am Main, Germany; 3 Chirurgisches Zentrum Zürich, Hirslanden Klinik, Zurich, Switzerland; 4 Chirurgie Zentrum Zentralschweiz Hirslanden Klinik St. Anna, Lucerne, Switzerland; 5 Adipositas und Stoffwechselzentrum, Klinik Hirslanden, Zurich, Switzerland; 6 Diabetes Adipositas Zentrum Zürich, Zollikerberg, Zurich, Switzerland; 7 Stoffwechselpraxis Zentralschweiz AG, Hirslanden Klinik St. Anna, Lucerne, Switzerland; 8 Present Address: Klinik für Viszeral- und Thoraxchirurgie, Kantonsspital Winterthur, Winterthur, Switzerland; 9 University of Zurich (UZH), Zurich, Switzerland

**Keywords:** Laparoscopic roux-en-Y gastric bypass, Roux limb, Weight loss, Bariatric surgery

## Abstract

**Purpose:**

The laparoscopic Roux-en-Y gastric bypass (LRYGB) is one of the standard procedures in metabolic surgery. Different limb lengths have been proposed in the past to maximize weight loss (WL) and reduce metabolic complications. Distal gastric bypass surgery with a very short common channel (CC) (up to 100 cm) has been often criticized due to frequent side effects such as malnutrition, bone weakening and short-bowel syndrome. We introduced a modified version of a distal LRYGB with a 50–70 cm long biliopancreatic limb (BPL) and an intermediate short CC (120–150 cm). Our primary goal was to compare the long-term WL between *distal* and *proximal* LRYGB in two cohorts of patients. Secondary outcomes were weight regain (WR), insufficient weight loss (IWL), postoperative complications and metabolic changes 5 years after surgery.

**Methods:**

In this retrospective study we collected data from 160 patients operated between 2014 and 2015, with a BMI of 37–44 Kg/m^2^. 101 patients underwent a *distal* and 59 patients a *proximal* LRYGB in two bariatric centers. WL was calculated as percent of excess of BMI loss (%EBMIL), loss of body mass index (Delta-BMI), percent of excess weight loss (%EWL) and percent of total weight loss (%TWL). Data were collected 3, 6, 9, 12, 24, 48 and 60 months after surgery.

**Results:**

The *distal* LRYGB resulted in significantly better 5-year-WL compared to the *proximal* bypass in terms of %EBMIL (median at 5 years: 83% vs. 65%, *p* = 0.001), %TWL (median at 5 years: 32% vs. 26%, *p* = 0.017) and %EWL (median at 5 years: 65% vs. 51%, *p* = 0.029), with equal major complications and metabolic alterations. In addition, WR was significantly lower in patients with *distal* bypass (18% vs. 35%, *p* = 0.032).

**Conclusions:**

*Distal* LYRGB with a 120–150 long CC results in better WL and WL-maintenance compared to *proximal* LRYGB without major side effects after five years.

**Supplementary Information:**

The online version contains supplementary material available at 10.1007/s00423-024-03348-2.

## Introduction

Gastric bypass surgery is still one of the most effective therapeutic options for severe obesity. Bypass surgery improves obesity-associated medical complications markedly [1–5] and therefore influences obesity-related mortality in a positive manor [6].

Since the first gastric bypass was performed in 1954, the procedure has been modified several times over the last decades. The increasing knowledge of the human physiology and endocrine system has shifted the attention from old concepts of “restriction” and “malabsorption” to the hormonal and metabolic mechanisms triggered by the duodenal and small bowel exclusion [7]. However, an adequate and endurable postoperative weight-loss (WL) remains a matter of great concern. Giving the fact that weight regain (WR) or an insufficient weight loss (IWL) are issues that most of the bypass-patients have to face on the long term [8–9], a greater focus has been set on the variations of the limb-lengths of gastric bypass surgery in order to create the most effective and durable results with the lowest rate of adverse events [10].

**Fig. 1:**
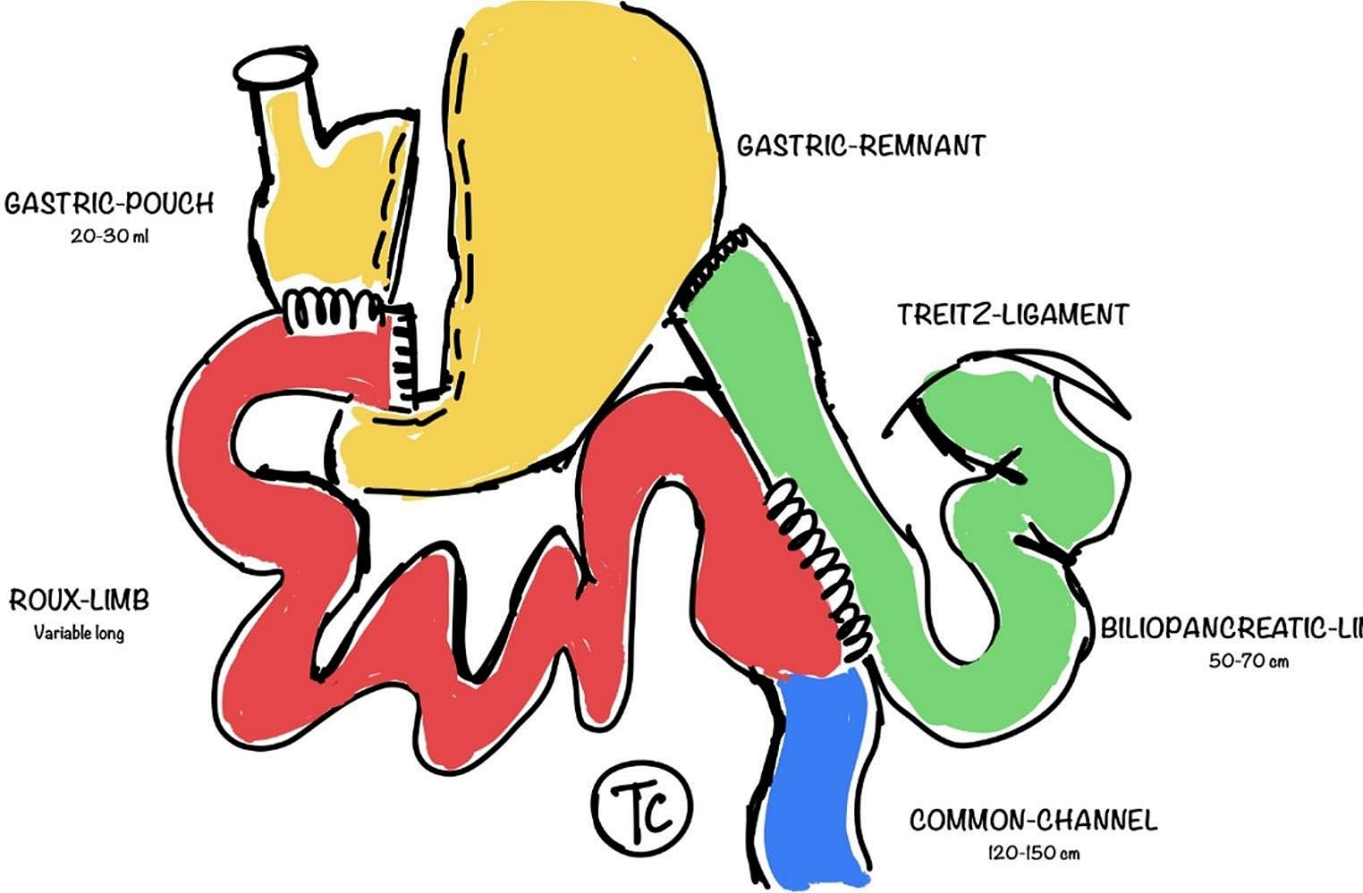
The laparoscopic *distal* Roux-en-Y Gastric Bypass (LRYGB) performed in our study

Various operations with different limb lengths have been developed in order to achieve a better and longer lasting WL, especially in patients with a BMI > 50 Kg/m^2^ [11–13], or to correct IWL or WR after previous bariatric-metabolic surgery [10–11, 14–15]. The *distal* LRYGB, the *very long limb* RYGB and the *biliopancreatic diversion* are just examples that serve these purposes by creating a very short common-channel (CC) (50–100 cm) [16–18]. However, these operations have been criticized for causing severe vitamin deficiencies, bone loss, protein malnutrition, decreased fat-absorption and short bowel syndrome [11, 19–22, 23]. Many bariatric centers have therefore no longer performed these operations due to these serious side effects. The laparoscopic *proximal* gastric bypass (LRYGB) with a short Roux limb has been adopted as a standard procedure in many centers over the last decades. However, WR and IWL have been an issue using this technique in the long term follow up. We therefore introduced a modified “*distal*” gastric bypass (Fig. 1) in our bariatric center in order to create an alternative to the classical distal LRYGB, with a fairer compromise between metabolic impact and maintenance of WL. Its definition matches with the Type II distal LRYGB described in the DISCOURSE-Trial for revisional surgery [24] and with the *very long roux limb* LRYGB cited in other studies [25–27], except for the length of the CC, which we defined between 120 and 150 cm from the ileocecal valve, similarly to Risstad et al. und Salte et al. [21–23], in order to have less problems with malabsorption.

We present our 5-year-results of *distal* gastric bypass compared to *proximal* gastric bypass surgery.

## Materials and methods

### Participants

We collected data from all patients that underwent a primary *distal* LRYGB between 2014 and 2015 in our bariatric center and analyzed them over 5 years. The data were compared to a cohort of patients that underwent a primary *proximal* LRYGB in the same time period in another bariatric center.

Inclusion criteria for the retrospective data collection were eligibility for bariatric-metabolic surgery according to the Swiss Society of Morbid Obesity (SMOB) and attendance to the regular follow-up visits within the first 5 years after surgery (at 3, 6, 9, 12, 24, 48 and 60 months). Patients that explicit declined their consent for data-collection were excluded from the analysis.

Our outcome reporting followed the guidelines of Brethauer et al. 2015 [28].

### Operative technique

All operations were carried out laparoscopically by four experienced bariatric surgeons.

The *distal* LRYGB consisted of an intermediate short CC (120–150 cm), a BPL of 50–70 cm and a variable long RL. The *proximal* LRYGB consisted of a RL of 150 cm, a BPL of 50–70 cm and a variable long CC. All operations were performed using the same technique. The first step was to create the gastric pouch using a three cartridges (60 mm) of a linear stapler (one horizontal and two vertical). The BPL was then measured from the ligament of Treitz and connected to the gastric pouch. The gastrojejunostomy was performed using a circular stapler in all patients in both cohorts. The insertion site of the circular stapler was closed and the jejunojejunostomy was separated from the gastrojejunostomy using a linear stapler (jejunal segment resection). In the *distal* LRYGB group, the CC was subsequently measured with a length of 120–150 cm from the ileocecal valve and then connected with the BPL using a linear-stapled jejunojejunostomy in all patients in both cohorts. In the *proximal* LRYGB group 150 cm of RL was measured from the gastrojejunostomy and anastomosed to the BPL. The open mesenteric defects (Petersen and Brolin) have been closed using a nonabsorbable suture in all *proximal* LRYGB patients. The closure of the intermesentery space (Brolin-space) was not routinely performed in the *distal* LRYGB cohort in order to avoid a damage to the blood-supply of the small bowel, due to the large size of the defect.

#### Primary end point

The primary end point of our study was weight loss (WL) 5 years after surgery. WL was calculated as percent of excess of BMI loss (%EBMIL), loss of body mass index (Delta-BMI), percent of excess weight loss (%EWL) and percent of total weight loss (%TWL) at 3, 6, 9, 12, 24, 48 and 60 months postoperative. The ideal body weight was set at the weight corresponding to a BMI of 25 Kg/m^2^.

We defined successful weight loss (SWL) as %EWL > 50 at 18 months after primary surgery, while a %EWL < 50 at 18 months postoperative was considered as insufficient weight loss (IWL) [29].

Due to the heterogeneous definition of weight regain (WR), we chose to calculate it using four of the six calculations suggested by Voorwinde et al. 2020 [30] (Supplementary Table 1). We excluded the statements “any weight gain” or “weight gain with a BMI > 35 Kg/m^2^ after a successful weight loss”, reported in the afore mentioned publication, as too approximate.

### Secondary end points

Further endpoints were postoperative complications and changes in the patients’ metabolic parameters after surgery, with particular focus on calcium-balance (parathormone, calcium, 25-OH-vitamin D), micronutrients (ferritin, albumin, zinc, vitamin B1 and vitamin B12) and hemoglobin A1c (HbA1c), especially in patients with type 2 diabetes or impaired glucose tolerance at baseline. All the laboratory measurements were collected at baseline and 5 years after surgery.

The complications were divided into major (serious, requiring intervention or re-operation) and minor (non-serious, requiring a conservative therapy or no therapy), as well as into early and late according to their onset (earlier or later than 30 days postoperative). Complications were also classified using the Clavien-Dindo Classification [31] and we calculated the Comprehensive Complication Index (CCI®) [32] for each patient having a complication within 30 days after surgery.

Patients’ information was collected from hospital or bariatric-centers database’s and recorded in a separate database using Microsoft® Excel® Spreadsheet Software, Version 16.16.27.

### Statistics

Descriptive data were expressed as proportions for categorical variables, means with standard deviation (SD) for parametric and medians with interquartile ranges (IQR) for non-parametric data, unless otherwise specified. Kolmogorov-Smirnov test was used to test distribution of data. For comparison, the students t-test was performed for parametric and the Mann-Whitney-test for non-parametric data. Fischer’s exact test was used for categorical variates. A p value of < 0.05 was considered significant. Dichotomizations are based on medians. Multivariable analysis was performed using stepwise regression for the endpoints > 78% EBMIL-5y, > 61% EWL-5y, > 30% TWL-5y and > 12 Kg/m^2^ Delta-BMI-5y.

Statistical analysis was performed with JMP® Software Version 14.1 (SAS Institute, Cary, USA). Graphics were made with Graph Pad Prism® 8.4.3 (Graph Pad Software, La Jolla, CA, USA). We performed a univariable and multivariable analysis to investigate the impact of the following six factors on our primary outcome: age, sex, baseline-BMI, obesity-related medical problems, operative technique and post-operative adverse events. Significant p value was set at < 0.05.

We could not perform a power analysis to improve the quality of our work, since numerous comparative studies of good quality, such as Müller et al. 2008 [16], Risstad et al. [22–23], Salminen et al. 2018 [33] and Peterli et al. 2018 [34], either did not achieve statistically significant results or did not show their data distribution.

This study was carried out in accordance with the Swiss Association of Research Ethics Committees, Section Zürich (BASEC-Nr. 2019–02477).

## Results

### Demographics

**Fig. 2:**
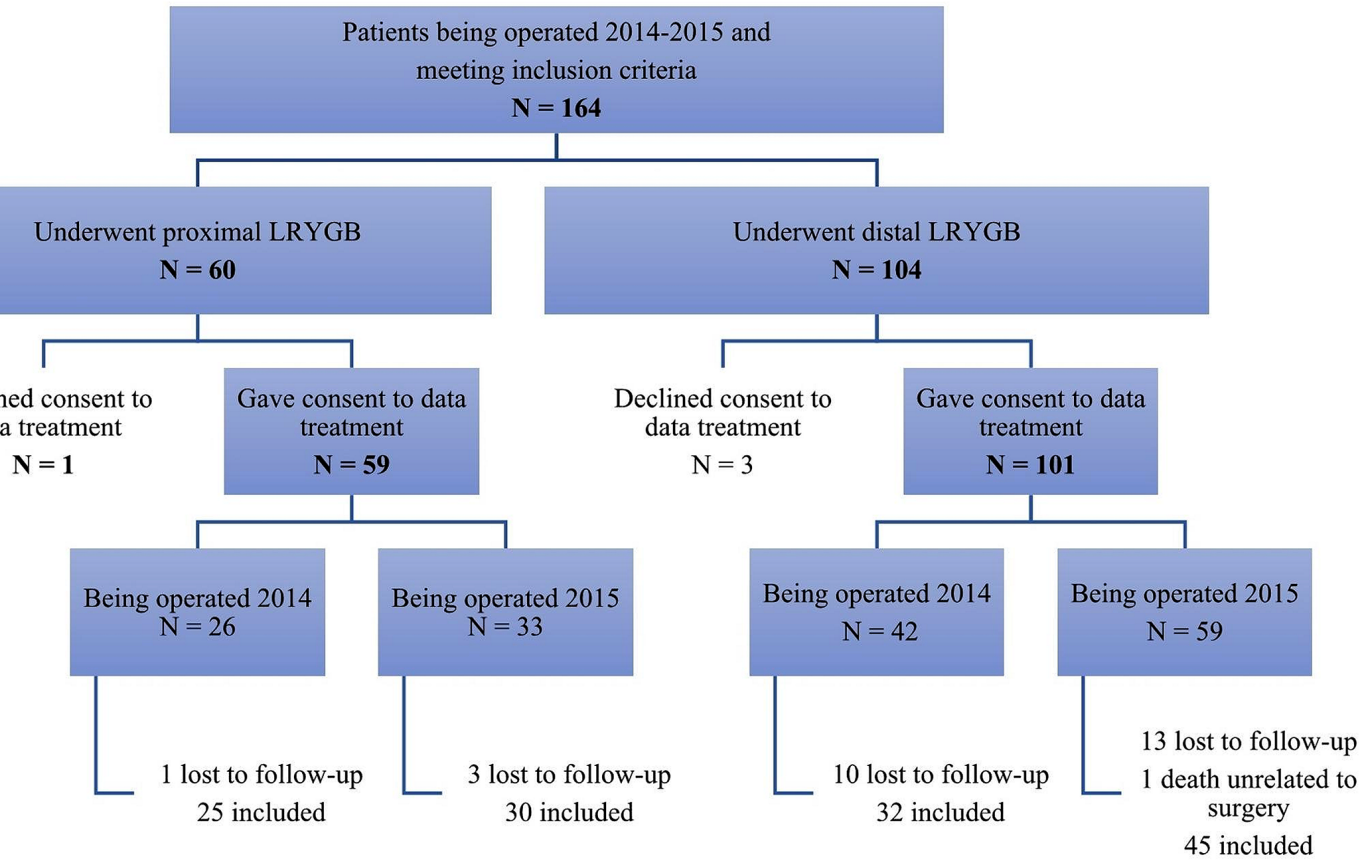
Study participants

One hundred and sixty-four patients underwent a *proximal* (*N* = 60) or *distal* (*N* = 104) LRYGB between January 2014 and December 2015 and were eligible for the retrospective data analysis. Four patients did not give their consent to the data collection und thus were excluded (one in the *proximal* and three in the *distal* group) (Fig. 2). Five years after surgery, we observed four lost-to-follow-up patients in the *proximal* and 23 in the *distal* LRYGB cohort. Only one patient died within five years after gastric bypass surgery in the *distal* LRYGB group due to a pancreatic cancer.

The baseline-characteristics of the two cohorts are given in Table 1. In both groups the majority were female (75% in the *proximal* vs. 78% in the *distal* LRYGB group) (*p* = 0.60). The median age was 45 (34–54) years in the *proximal* LRYGB group and 47 (39–55) years in the *distal* cohort (*p* = 0.18). At baseline, thirty-six patients (61%) had a BMI > 40 Kg/m^2^ in the *proximal* and 49 (49%) in the *distal* LRYGB group (*p* = 0.13).

**Table 1:** Demographics

	Proximal LRYGB (*n* = 59)	Distal LRYGB (*n* = 101)	*p*
Gender Female, *n* (%) Male, *n* (%)	44 (75%) 15 (25%)	79 (78%) 22 (22%)	0.60
Age, median (IQR)	45 (34–54)	47 (39–55)	0.18
Body Mass Index (BMI), at baseline, mean	42 (38–44)	40 (37–44)	0.39
BMI 35-39.9 Kg/m^2^	22 (37%)	42 (42%)	0.60
BMI > 40 Kg/m^2^	36 (61%)	49 (49%)	0.13
Obesity-related medical problems, n (%)	59 (100%)	55 (55%)	0.99
Arterial hypertension, n (%)	25 (42%)	31 (31%)	0.14
Diabetes mellitus Impaired glucose tolerance	12 (20%) 32 (54%)	12 (12%) 26 (26%)	< 0.001
Sleep apnea	16 (27%)	12 (12%)	0.02
Dyslipidemia	37 (63%)	24 (24%)	< 0.001
Bone changes	54 (92%)	6 (6%)	< 0.001
Hyperandrogenism	1 (2%)	0	0.99
Polycystic ovary	1 (2%)	1 (1%)	0.70
Hyperuricemia	15 (25%)	9 (9%)	0.006
Previous abdominal surgery	26 (44%)	48 (48%)	0.67

There was no significant difference in the overall incidence of obesity-related medical problems (*p* = 0.99) or in the rate of previous abdominal surgery (*p* = 0.67) between the two cohorts. However, the distribution of diabetes mellitus / impaired glucose tolerance, dyslipidemia and bone changes were more frequent in patients that underwent a *proximal* gastric bypass (*p* < 0.001).

### Missing data

The BW and BMI measures 5 years after surgery were available for 55 from 59 patients after *proximal* LRYGB and for 77 from 101 patients after *distal* LRYGB.

The data to assess SWL and IWL at 18 months after surgery were complete for the *proximal* LRYGB cohort (59/59), but not for the *distal* group (92/101 patients).

Among the laboratory findings we had some missing data since blood sampling was not standardized before and after surgery. In addition to that, we could not compare Vitamin B12 levels in the two cohorts, since it was expressed as whole in the *proximal* group and as active Vitamin B12 (Holotranscobalamin) in the *distal* cohort.

### Weight loss

**Fig. 3:**
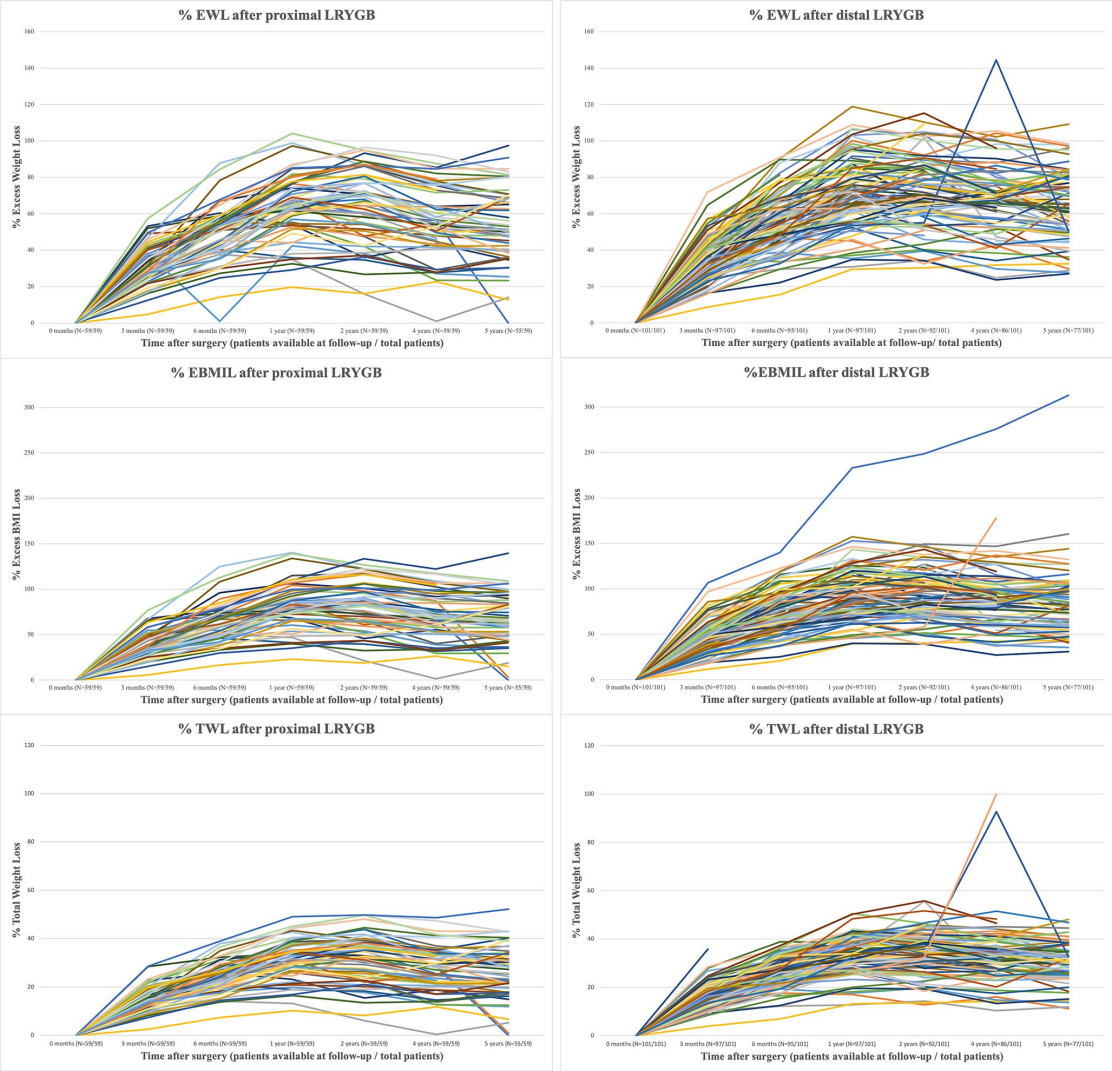
Weight loss *proximal* vs. *distal* LRYGB

Our weight loss results are shown in Table 2; Fig. 3.

**Table 2:** Weight Loss (WL), Weight Regain (WR) and Insufficient Weight Loss (IWR)

	Proximal LRYGB (*n* = 59)	Distal LRYGB (*n* = 101)	*p*
**Weight Loss (WL)**			
Delta-BMI at 5 years (*median (IQR*))	11 (8–14)	13 (10–15)	0.09
%TWL at 5 years	26 (20–34)	32 (26–35)	0.017
%EBMIL at 5 years	65 (48–88)	83 (61–103)	0.001
%EWL at 5 years	51 (39–69)	65 (51–79)	0.029
**Weight Regain (WR)**			
Increase of > 10 Kg from nadir body weight, n (%)	19/55 (35%)	14/77 (18%)	0.032
Increase of > 25% EWL from nadir	5/55 (9%)	10/77 (13%)	0.49
Increase in BMI of 5 Kg/m^2^ from nadir	7/55 (13%)	8/77 (10%)	0.68
Increase of > 15% of body weight at nadir	16/55 (29%)	11/77 (14%)	0.038
**Insufficient weight loss (IWR)**			
%EWL < 50% at 18 months after surgery, n (%)	15/59 (25%)	12/92 (13%)	0.053

The *distal* LRYGB resulted in significantly better long-term results than the *proximal* gastric bypass in terms of %EBMIL (median at 5 years: 83% vs. 65%, *p* = 0.001), %TWL (median at 5 years: 32% vs. 26%, *p* = 0.017) and %EWL (median at 5 years: 65% vs. 51%, *p* = 0.029). 5-years-Delta-BMI between the two cohorts was not significant (13 vs. 11, *p* = 0.09).

We observed a significantly higher rate of long-term WR after *proximal* compared to the *distal* LRYGB, according to WR-definition 1 (35% vs. 18%, *p* = 0.032) and WR-definition 4 (29% vs. 14%, *p* = 0.038).

Patients in the *proximal* LRYGB group were more likely to face IWL 18 months after surgery compared to the *distal* LRYGB group, although the difference was not significant (25% vs. 13%, *p* = 0.053).

The multivariable analysis (Table 3) showed a significant correlation between “female sex” and %EBMIL > 78 (p < 0.001), %EWL > 61 (p</=0.001), %TWL > 30 (p < 0.02) at 5 years postoperative, but not between “female sex” and long-term Delta-BMI > 12 Kg/m^2^ (p = 0.06). The impact of the operative technique (*distal* vs. *proximal* LRYGB) was only significant on the Delta-BMI at 5 years (p = 0.049). The presence of „obesity-associated medical problems at baseline” showed a positive correlation with long-term %EBMIL in the univariable (*p* = 0.013) but did not remain significant in the multivariable analysis (*p* = 0.54).

**Table 3:** Multivariable analysis (a-d)

**(a) Multivariable Analysis for the endpoint > 78% EBMIL-5y**														
**Analysis**		**univariate**						**multivariate**						
Variate		Odds ratio (CI)			p-value			Adjusted OR (CI)			p-value			
Age > 47 years vs. < 47 years		0.51 (0.26–1.02)			0.07			0.49 (0.22–1.11)			0.09			
Postoperative complications (major complications ≥IIIA)		0.76 (0.32–1.77)			0.52			0.41 (0.16–1.07)			0.07			
Obesity-related medical problems yes vs. no		0.36 (0.16–0.81)			**0.013**			0.53 (0.18–1.54)			0.24			
Distal LRYGB vs. Proximal LRYGB		2.28 (1.12–4.62)			**0.023**			1.83 (0.72–4.65)			0.21			
Sex female vs. male		11.23 (3.17–39.76)			**< 0.001**			12.96 (3.46–48.62)			**< 0.001**			
**(b) Multivariable Analysis for the endpoint > 61% EWL-5y**														
**Analysis**		**univariate**						**multivariate**						
Variate		Odds ratio (CI)			p-value			Adjusted OR (CI)			p-value			
Age > 47 years vs. < 47 years		0.65 (0.33–1.30)			0.22			0.68 (0.30–1.52)			0.35			
Postoperative complications (major complications ≥IIIA)		0.76 (0.32–1.77)			0.52			0.43 (0.17–1.10)			0.08			
Obesity-related medical problems yes vs. no		0.43 (0.20–0.94)			0.032			0.55 (0.19–1.62)			0.28			
Distal LRYGB vs. Proximal LRYGB		2 (0.99–4.04)			0.053			1.55 (0.61–3.95)			0.36			
Sex female vs. male		18.29 (4.10-81.47)			**< 0.001**			20.55 (4.47–94.59)			**< 0.001**			
**(c) Multivariable Analysis for the endpoint > 30% TWL-5y**														
**Analysis**		**univariate**						**multivariate**						
Variate		Odds ratio (CI)			p-value			Adjusted OR (CI)			p-value			
Age > 47 years vs. < 47 years		0.61 (0.31–1.22)			0.16			0.64 (0.31–1.35)			0.24			
Postoperative complications (major complications ≥IIIA)		0.95 (0.41–2.21)			0.90			0.69 (0.28–1.69)			0.41			
Obesity-related medical problems yes vs. no		0.48 (0.22–1.05)			0.06			0.71 (0.27–1.87)			0.48			
Distal LRYGB vs. Proximal LRYGB		1.90 (0.94–3.83)			0.07			1.60 (0.68–3.79)			0.29			
Sex female vs. male		3.30 (1.27–8.46)			**0.01**			3.19 (1.20–8.47)			**0.02**			
**(d) Multivariable Analysis for the endpoint Delta-BMI 5 years > 12 kg/m** ^**2**^														
**Analysis**		**univariate**						**multivariate**						
Variate	Odds ratio (CI)			p-value			Adjusted OR (CI)			p-value				
Age > 47 years vs. < 47 years	0.69 (0.35–1.38)			0.30			0.66 (0.32–1.39)			0.27				
Postoperative complications (major complications ≥IIIA)	1.14 (0.49–2.66)			0.76			0.93 (0.38–2.27)			0.88				
Obesity-related medical problems yes vs. no	0.66 (0.31–1.41)			0.28			1.26 (0.48–3.29)			0.64				
Distal LRYGB vs. Proximal LRYGB	2.16 (1.07–4.38)			**0.033**			2.39 (1.00-5.69)			**0.049**				
**Sex female vs. male**	2.62 (1.05–6.54)			**0.040**			2.48 (0.96–6.37)			0.06				

13 patients after *proximal* and 25 patients after *distal* LRYGB experienced major postoperative adverse events (Table 4) with a total of 18 major complications in the *proximal* and 26 in the *distal* group. Most of them occurred later than 30 days after surgery (12 after *proximal* and 21 after *distal* LRYGB). The median CCI for complications within 30 days was 33.7 (24–42) in the *proximal* LRYGB group and 20.9 (21–42) in the *distal* LRYGB cohort (*p* = 0.25).

**Table 4:** Postoperative adverse events

	Proximal LRYGB (*n* = 59 patients)	Distal LRYGB (*n* = 101 patients)	*p*
*Postoperative adverse events*			
Major complications, N	18 (in 13 patients)	26 (in 25 patients)	0.67
Within 30 days after surgery	6 (in 3 patients)	5 (in 5 patients)	0.97
At more than 30 days after surgery	12 (in 11 patients)	21 (in 20 patients)	0.86
Minor complications, N	42 (in 32 patients)	45 (in 40 patients)	0.07
Within 30 days after surgery	4 (in 3 patients)	7 (in 7 patients)	0.64
At more than 30 days after surgery	38 (in 29 patients)	38 (in 35 patients)	0.07
CCI Within 30 days after surgery	33.7 (24–42)	20.9 (21–42)	0.25

Supplementary Table 2 shows the adverse events in detail. The most common serious complication was “internal hernia” (7 cases in the *proximal* and 17 in the *distal* cohort, *p* = 0.40).

According to the serum values displayed in Table 5, none of our patients showed a severe long-term hypoalbuminemia (Albumin < 30 g/l). Improvement in HbA1c (HbA1c < 6% after being pathological at baseline) was demonstrated in 7 out of 9 patients after *proximal* and in 10 out of 16 patients after *distal* LRYGB (*p* = 0.43). In the *distal* LRYGB group, significantly more patients had an increased parathyroid hormone level (PTH, > 6.9 pmol/l) than the proximal cohort at 5 years post-surgery (59/96 vs. 13/59, *p* < 0.001), but the two cohorts did not show a significant difference in the prevalence of secondary hyperparathyroidism (PTH > 6.9 pmol/l and calcium < 2.2 mmol/l) (12% vs. 8%, *p* = 0.65).

**Table 5:** Blood serum measurements

	Proximal LRYGB (*n* = 59)	Distal LRYGB (*n* = 101)	*p*
*Blood serum measurements*			
Albumin < 30 g/l at 5 years	0/59	0/95	
A1C < 6% at 5 years in patients, with A1C > 6% at baseline	7/9 (78%)	10/16 (63%)	0.43
PTH > 6.9 pmol/l at 5 years	13/59 (22%)	59/96 (62%)	**< 0.001**
Patients with a PTH > 6,9 pmol/l and Calcium < 2.2 mmol/l	1/13 (8%)	7/58 (12%)	0.65

## Discussion

Our long-term results of *distal* gastric bypass in patients with a BMI between 37 and 44 kg/m^2^ are encouraging. In comparison to *proximal* laparoscopic Roux-en-Y gastric bypass, the *distal* LRYGB resulted in a significantly better %EBMIL, %EWL and %TWL, without a notable increase in adverse events or metabolic problems five years after surgery. Our study findings differ from those of other similar studies, specifically the conclusions drawn by Müller et al. (2008) [16] and Risstad et al. / Salte et al. (2016, 2021) [22–23], which evaluated BMI-decrease and did not find a significant difference between the two techniques. The missing difference in the latter study [22–23] might be explained by the inclusion of patients with BMI higher than 50 kg/m^2^. Delta-BMI and %TWL depend less on the initial BMI, while %EWL heavily does. Our Delta-BMI analysis did not yield significant results, emphasizing the limitations of this parameter in accurately reporting WL. This finding reinforces the vital need for standardization in reporting outcomes for bariatric-metabolic surgery, which is essential to maintain credibility and enable consistent comparisons across different studies.

In accordance with our results, the rate of adverse events in the above-mentioned studies [16, 22–23] was not significantly different. Our primary concern after *distal* LRYGB surgery, was the incidence of internal hernia, since we did not consistently close Brolin’s space. Internal hernias were indeed the most frequent major complication in the *distal* LRYGB group but the incidence between the two cohorts was not significantly different (*p* = 0.40). The high incidence of internal hernias in both groups, if compared with the current literature [16, 18, 20, 33–35], underlines the need for a more careful closure of the mesenteric defects.

Fortunately, we did not observe any cases of severe malnutrition, gastrointestinal side effects or other significant metabolic issues in the *distal* gastric bypass group. This might be due to the length of the CC, which was set at 120–150 cm in this study. However, we only reported 5-year follow up data and potential side effects can occur in a later stage. Our data revealed a 5-year prevalence rate of 62% for altered parathyroid hormone (PTH) levels in the *distal* LRYGB cohort, which was significantly higher than the 22% observed in the *proximal* group (*p* < 0.001). This finding indicates the need for a careful monitoring of calcium metabolism in these patients to prevent the onset of secondary hyperparathyroidism. We have to consider that severe malnutrition and metabolic complications often occur later than five years after the operation. Therefore, we cannot conclude that this procedure without any risk on the long term.

The significantly higher prevalence of diabetes mellitus/impaired glucose tolerance observed at baseline in our *proximal* LRYGB cohort compared to the *distal* LRYGB cohort (20%/54% vs. 12%/26%, *p* < 0.001) might be seen as a bias, suggesting that these patients had a worse predisposition to lose weight. However, our long-term WL results for this cohort were comparable to other studies, including Nergaard et al. 2014 [35] and Peterli et al. 2018 [34] (WL reported as %EBMIL) or Zhang et al. 2014 [36] and Salminen et al. 2018 [33–36] (WL reported as %EWL) and our multivariate analysis showed that the impact of obesity-associated medical problems on WL was not significant.

This work has clearly the limitations of a retrospective, non-matched cohort study. Despite our best efforts to minimize reporting and collection biases by strictly adhering to the guidelines for standardized outcome reporting in bariatric surgery suggested by Brethauer et al. [28] and performing a multivariable analysis, we cannot rule out the possibility of information bias due to the retrospective design and the presence of some missing data. Nevertheless, we believe that this study provides important information for the bariatric-metabolic surgery community. Our evidence supports some ongoing prospective studies like the DUCATI study [25–27] in considering a primary very long Roux-limb gastric bypass as a superior alternative to the standard LRYGB in order to achieve a more adequate and long-lasting WL. Elongation of the CC together with a long RL, while keeping the BPL constant, as we did in our procedure, might be critical in mitigating issues related to malabsorption and thus reduce the skepticism regarding the *distal* LRYGB. A primary *distal* gastric bypass operation may be technically more challenging than the standard *proximal* gastric bypass, but could play an important role in a patient-tailored approach that addresses diverse needs and objectives within bariatric-metabolic surgery. Furthermore, we could only provide five-year data of our patients due to the loss of follow up and the study design. However, obesity affects patients over decades. Weight regain and metabolic/malnutritive complications can occur a later stage ten years after the operation. Longer follow up data would be of great value and might alter our results. Better evidence with longer follow up periods is needed to support these considerations.

## Conclusion

Our findings suggest that a primary modified *distal* laparoscopic Roux-en-Y gastric bypass (LYRGB) procedure with a common channel length of 120–150 cm and a biliopancreatic limb of 50–70 cm is a feasible and safe therapeutic option. It appears to result in superior long-term weight loss and weight loss maintenance compared to the standard *proximal* LYRGB technique. The metabolic complications and adverse events five years after surgery are reasonable.

### Electronic supplementary material

Below is the link to the electronic supplementary material.

Supplementary Material 1

Supplementary Material 2

## Data Availability

The authors confirm that the data supporting the findings of this study are available within the article and its supplementary materials. Restrictions apply to the availability of the raw data, which were used under license for this study. These Data are available with the permission of the Swiss Association of Research Ethics Committees, Section Zürich.
