# Supplementary material for: Long-term weight loss of distal gastric bypass is moderately superior compared to proximal gastric bypass in patients with a BMI of 37–44 Kg/m2
Source: Langenbecks Arch Surg. 2024 May 21;409(1):162. doi: 10.1007/s00423-024-03348-2 (PMC11108920; doi:10.1007/s00423-024-03348-2)
Supplement: Supplementary file 1 — Supplementary Material 1 [file 423_2024_3348_MOESM1_ESM.docx]

**Supplementary Table 1: Definitions of WR with formulas ^(2)^**

| An increase of > 10 Kg from weight at nadir | (Body Weight at 5y – Body Weight at nadir) > 10 Kg |
| --- | --- |
| An increase of > 25% EWL from  EWL at nadir | (EWL at nadir – EWL at 5y) > 25 |
| An increase in BMI of 5 Kg/m^2^ | (BMI at 5y – BMI at nadir) > 5 |
| An increase of > 15% of total body weight at nadir | (Body weight in kg at 5y – Body weight in Kg at nadir) / Body weight at nadir in Kg) *100 > 15 |
