# Supplementary material for: Long-term weight loss of distal gastric bypass is moderately superior compared to proximal gastric bypass in patients with a BMI of 37–44 Kg/m2
Source: Langenbecks Arch Surg. 2024 May 21;409(1):162. doi: 10.1007/s00423-024-03348-2 (PMC11108920; doi:10.1007/s00423-024-03348-2)
Supplement: Supplementary file 2 — Supplementary Material 2 [file 423_2024_3348_MOESM2_ESM.docx]

**Supplementary Table 2: Postoperative adverse events in detail**

|  | **Proximal**  **Gastric Bypass**  **(n=59 patients)** | **Distal**  **Gastric Bypass (n=101 patients)** | **p** |
| --- | --- | --- | --- |
| **Major complications within 30 days after surgery, N** | 6 (in 3 patients) | 5 (in 5 patients) | 0.97 |
| Internal bleeding | 0 | 0 |  |
| Gastrointestinal bleeding | 1 | 1 | 0.70 |
| Leak | 0 | 0 |  |
| Stenosis | 1 | 0 | 0.19 |
| Small bowel obstruction | 1 | 2 | 0.90 |
| Bowel perforation | 0 | 0 |  |
| Surgical site infection | 0 | 1 | 0.44 |
| Incisional hernia | 1 | 0 | 0.19 |
| Internal hernia | 0 | 0 |  |
| Marginal ulcers | 0 | 0 |  |
| Gastric remnant distention | 0 | 0 |  |
| Venous thrombotic event | 0 | 0 |  |
| Heart infarction | 0 | 0 |  |
| Cerebral vascular infarction | 0 | 0 |  |
| Renal failure | 0 | 0 |  |
| Respiratory failure | 2 | 1 | 0.28 |
| Chronic nausea and vomiting | 0 | 0 |  |

|  | **Proximal**  **Gastric Bypass**  **(n=59 patients)** | **Distal**  **Gastric Bypass**  **(n=101 patients)** | **p** |
| --- | --- | --- | --- |
| **Major complications at more than 30 days after surgery, N** | 12 (in 11 patients) | 21 (in 20 patients) |  |
| Internal bleeding | 0 | 0 |  |
| Gastrointestinal bleeding | 0 | 0 |  |
| Leak | 0 | 0 |  |
| Stenosis | 0 | 0 |  |
| Small bowel obstruction | 2 | 2 | 0.59 |
| Bowel perforation | 1 | 0 | 0.19 |
| Surgical site infection | 0 | 0 |  |
| Incisional hernia | 1 | 1 | 0.70 |
| Internal hernia | 7 | 17 | 0.40 |
| Marginal ulcers | 0 | 1 | 0.44 |
| Gastric remnant distention | 0 | 0 |  |
| Venous thrombotic event | 1 | 0 | 0.19 |
| Heart infarction | 0 | 0 |  |
| Cerebral vascular infarction | 0 | 0 |  |
| Renal failure | 0 | 0 |  |
| Respiratory failure | 0 | 0 |  |
| Chronic nausea and vomiting | 0 | 0 |  |

|  | **Proximal**  **Gastric Bypass**  **(n=59 patients)** | **Distal**  **Gastric Bypass (n=101 patients)** | **p** |
| --- | --- | --- | --- |
| **Minor complication within 30 days after surgery, N** | 4 (in 3 patients) | 7 (in 7 patients) | 0.64 |
| Internal bleeding | 1 | 3 | 0.62 |
| Gastrointestinal bleeding | 1 | 1 | 0.70 |
| Leak | 0 | 1 | 0.44 |
| Stenosis | 0 | 0 |  |
| Small bowel obstruction | 0 | 1 | 0.44 |
| Surgical site infection | 0 | 0 |  |
| Incisional hernia | 0 | 1 | 0.44 |
| Marginal ulcers | 1 | 0 | 0.19 |
| Acute renal failure | 0 | 0 |  |
| Dehydration | 0 | 0 |  |
| Nausea and vomiting | 0 | 0 |  |
| Nephrolithiasis | 0 | 0 |  |
| Reflux | 1 | 0 | 0.19 |
| Short bowel syndrome | 0 | 0 |  |
| Cholecystolithiasis | 0 | 0 |  |
| Urinary tract infection | 0 | 0 |  |

|  | **Proximal**  **Gastric Bypass**  **(n=59 patients)** | **Distal**  **Gastric Bypass (n=101 patients)** | P |
| --- | --- | --- | --- |
| **Minor complications at more than 30 days after surgery, N** | 38 (in 29 patients) | 38 (in 34 patients) | 0.07 |
| Internal bleeding | 0 | 0 |  |
| Gastrointestinal bleeding | 0 | 0 |  |
| Leak | 0 | 0 |  |
| Stenosis | 1 | 1 | 0.70 |
| Small bowel obstruction | 2 | 1 | 0.28 |
| Surgical site infection | 0 | 0 |  |
| Incisional hernia | 0 | 0 |  |
| Marginal ulcers | 4 | 6 | 0.83 |
| Acute renal failure | 0 | 0 |  |
| Dehydration | 0 | 0 |  |
| Nausea and vomiting | 0 | 0 |  |
| Nephrolithiasis | 6 | 4 | 0.12 |
| Cholecystolithiasis | 6 | 22 | 0.06 |
| Urinary tract infection | 1 | 0 | 0.19 |
